# Surgery Versus Ultrasound-Guided Foam Sclerotherapy for the Treatment of Isolated Anterior Saphenous Vein Incompetence: A Single-Center Randomized Trial

**DOI:** 10.3390/jcm15218343

**Published:** 2026-10-26

**Authors:** Philip Kienzl, Hannah Lorenz, Sabine Stolkovich, Harald Kittler, Kornelia Böhler, Stanislava Tzaneva

**Affiliations:** Department of Dermatology, Medical University of Vienna, Währinger Gürtel 18-20, A-1090 Vienna, Austriastanislava.tzaneva@meduniwien.ac.at (S.T.)

**Keywords:** anterior accessory saphenous vein, anterior saphenous vein, ultrasound-guided foam sclerotherapy, high ligation, phlebectomy, randomized controlled trial

## Abstract

**Background:** Isolated anterior saphenous vein (ASV) incompetence is a clinically relevant source of symptomatic anterior thigh varicose veins, which may cause pain, heaviness, swelling and serious complication such as superficial vein thrombosis. Therefore, targeted management is often required, yet evidence guiding treatment remains limited. This single-center, randomized trial compared high ligation surgery in combination with phlebectomy and ultrasound-guided foam sclerotherapy (UGFS). **Methods:** Thirty-nine limbs of 35 patients (19 allocated to surgery, 20 to UGFS) were treated and followed up at 1 and 3 years. The primary outcome was ASV-territory recurrence defined as reflux time > 0.5 s along the ASV in the UGFS group or new anterior thigh varicosities connected to the previous saphenofemoral junction in the surgical group at 3 years. Secondary outcomes were the same duplex endpoint at 1 year, recurrence of clinically visible varicose veins, and groin neovascularization at 1 and 3 years. **Results:** ASV-territory recurrence was numerically higher after UGFS (63% vs. 33%) at 3 years, although the difference was not statistically significant, whereas recurrence was higher after UGFS than surgery at 1 year (65% vs. 11%; *p* < 0.001). Clinical recurrence rates did not differ significantly between groups at 1 and 3 years. Neovascularization was observed exclusively in the surgery group at 1 year and remained more frequent at 3 years (44% vs. 5%). **Conclusions:** Our findings suggest better short-term disease control with surgery than with UGFS for isolated ASV incompetence, whereas the prespecified primary endpoint of ASV-territory recurrence at 3-years did not differ significantly between groups. Both surgery and UGFS may therefore be considered for selected patients, with treatment choice guided by patient-centered factors, including vein anatomy, invasiveness, and patient preference.

## 1. Introduction

The incompetent anterior saphenous vein (ASV) [1] is an important source of symptomatic venous disease. Contemporary series and reviews suggest that isolated ASV incompetence is present in approximately 10–14% of patients [2,3]. Furthermore, it is frequently identified as a source of recurrent venous disease after prior truncal interventions [4]. Previous studies have shown that ASV incompetence may cause disease severity broadly comparable to that observed in great saphenous vein (GSV) incompetence, including similar clinical manifestations such as pain, heaviness, visible varicose veins, edema, and skin changes [3,5]. Multiple therapeutic strategies can be used for ASV incompetence, including conventional surgery (high ligation with/without short stripping) [6], endovenous thermal ablation (EVTA) [7,8], cyanoacrylate closure [9], mechanochemical ablation [10] as well as ultrasound-guided foam sclerotherapy (UGFS) [11]. EVTA uses thermal energy to close the vein, cyanoacrylate closure relies on an adhesive, and mechanochemical ablation combines mechanical injury with chemical sclerosis, with all three techniques requiring cannulation of the target vein. Current North American and European guidelines favor EVTA when feasible [12,13].

However, treatment of ASV incompetence can be challenging due to vein characteristics, such as variable vein diameter, short intrafascial course, tortuosity, distance to the superficial femoral artery, and association with refluxing veins in inguinal lymph nodes, which may complicate cannulation for endovenous treatment [4]. In contrast, both surgery and UGFS can be applied across a wide range of anatomical ASV variations. Furthermore, practical considerations, including operator experience and familiarity with treatment methods, influence treatment selection. In addition, a patient-centered approach that incorporates clinical presentation, patient anatomy, and treatment goals is essential [12].

Surgical treatment, which has been practiced for decades, benefits from extensive clinical experience. When performed appropriately, it may allow precise removal of the source of incompetence without leaving a residual stump and does not require special patient selection.

Evidence from randomized controlled trials addressing isolated ASV incompetence is limited, and most available data are derived from observational studies, mixed-vein cohorts, or studies with limited ASV-specific outcome reporting [11,14,15]. Therefore, clarification of the effectiveness of surgical treatment versus UGFS for the treatment of ASV incompetence remains clinically relevant, particularly as both modalities continue to be established treatment options in contemporary phlebologic practice.

We hypothesized that, in patients with isolated ASV incompetence, high ligation with phlebectomy would result in a lower rate of ASV-territory recurrence at 3 years than UGFS, according to the predefined treatment-specific duplex criteria.

## 2. Methods

This was an investigator-initiated, unblinded, prospective, single-center, randomized clinical trial conducted at the Department of Dermatology, Medical University of Vienna. The study compared high ligation surgery with phlebectomy under local anesthesia versus UGFS for the treatment of isolated ASV incompetence. Follow-up was performed at 1 year to evaluate short-term outcome and at 3 years to assess mid-term outcome, consistent with the International Union of Phlebology (UIP) consensus document [16]. The study protocol was approved by the institutional ethics committee (ethics number 255/2006; approval date: June 2006). The trial was retrospectively registered at ClinicalTrials.gov (NCT02010723; date: November 2013), conducted in accordance with the Declaration of Helsinki, and the principles of Good Clinical Practice. Written informed consent was obtained from all participants before inclusion.

All evaluations were performed by clinical examination and duplex ultrasound in the standing position using a 6-MHz linear probe (Logiq 7, General Electric, Milwaukee, WI, USA). Vein incompetence was assessed by manual thigh compression/decompression and the Valsalva maneuver and defined as reverse flow >0.5 s. The maximal diameter of the treated intrafascial ASV and its direct extrafascial continuation, excluding further tributaries, was recorded without restriction to a predefined distance from the saphenofemoral junction [17].

## 3. Patients

Patients aged 18–75 years with isolated, duplex-confirmed ASV incompetence (reflux time > 0.5 s) and varicose tributaries were eligible. Additional inclusion criteria were written informed consent and ability to comply with follow-up. Patients with concomitant GSV incompetence, axial deep venous reflux following deep vein thrombosis, acute deep or superficial venous thrombosis, hypercoagulability, allergy to polidocanol or local anesthetics, immobility, patent foramen ovale, local skin infection, pregnancy, breast feeding, and peripheral arterial occlusive disease (Fontaine stage III–IV) were excluded. Thirty-nine limbs from 35 patients were randomized in a 1:1 ratio to surgery or UGFS at the limb level. In patients with bilateral eligible ASV incompetence, each limb was randomized independently. The random allocation sequence was generated by a statistician and implemented using sequentially numbered, opaque, sealed envelopes. Envelopes were opened only after written informed consent had been obtained. Participants were enrolled before the allocation envelope was opened, and the investigators involved in enrolment and treatment had no access to the allocation sequence before assignment. Data were recorded on case report forms and monitored by certified dermatologists.

## 4. Interventions

### 4.1. Surgery

After infiltration with tumescent local anesthesia, high ligation was performed through an incision approximately 2–3 cm above the inguinal fold. The procedure included division of all tributaries at the saphenofemoral junction, intentional flush ligation of the GSV as part of the conventional high-ligation technique despite baseline GSV competence, andy electrocoagulation of the exposed endothelium.

The major part of the ASV in its saphenous compartment was removed via the groin incision without using a vein stripper. More distally located parts of the ASV as well as tributaries were removed via multiple stab incisions using Várady phlebectomy hooks and fine tipped mosquito clamps. Wounds were closed in layers and sterile dressings were applied. The patients were instructed to wear class II compression stockings (23–32 mmHg) for 2–3 weeks until resorption of hematoma.

### 4.2. Ultrasound-Guided Foam Sclerotherapy

UGFS was performed with polidocanol (Aethoxysklerol^®^, Chemische Fabrik Kreussler & Co. GmbH, Wiesbaden, Germany) at concentrations of 0.5–3%. Foam was prepared using the Tessari method using two disposable syringes and a three-way tap, at a liquid-to-air ratio of 1:4. The ASV and varicose tributaries were located by ultrasound, punctured, and freshly prepared foam was instilled. Class II compression stockings (23–32 mmHg) were worn immediately post-procedure for up to 3 weeks. During the initial treatment phase after study inclusion, UGFS was repeated at weekly intervals, up to a maximum of three sessions, until closure of the ASV was achieved. Foam volumes were converted to liquid sclerosant equivalents and polidocanol dose (mg) was calculated as concentration (%) × 10 × liquid (mL). For each date, doses from multiple injections were summed.

After completion of the scheduled 1-year clinical and duplex follow-up assessment, UGFS retreatment was permitted when clinically indicated, defined as clinically visible ASV-territory recurrence and the patient’s request for additional treatment. Recurrence detected at the 1-year visit was recorded before any additional treatment was performed. No further interventions were performed between the 1-year and 3-year follow-up visits.

### 4.3. Outcomes

For the assessment of outcomes, clinical as well as duplex-sonographic evaluation was performed as described before. The primary outcome was duplex-sonographic recurrence in the ASV territory at 3 years in both groups. However, the anatomical manifestation of recurrence was necessarily treatment-specific and therefore defined as reverse blood flow of >0.5 s along the ASV and its tributaries in the sclerotherapy group or in new anterior thigh varicosities connected to the previous saphenofemoral junction (NATV) in the surgical group at 3 years. Secondary outcomes included persistent or recurrent reflux in the same territory at 1 year, clinical recurrence, defined as new visible varicose veins in the treated ASV territory, and neovascularization (grade 0 [no new veins], grade 1 [new veins < 4 mm], grade 2 [new veins ≥ 4 mm]) [18,19] at 1 and 3 years. New-onset GSV incompetence (reflux time > 0.5 s) at 1 and 3 years was assessed as an exploratory variable, whereas symptoms, Clinical–Etiological–Anatomical–Pathophysiological (CEAP) classification, and medication history were recorded descriptively in the present analysis.

### 4.4. Statistical Analysis

All analyses were performed according to the intention-to-treat principle and each limb was analyzed independently. Two sensitivity analyses were performed: one excluding patients with bilateral treatment and another classifying additional UGFS retreatment as treatment failure. Adverse events were not captured in a standardized analyzable study database and are therefore not reported as comparative outcomes. Missing follow-up data were not imputed, and analyses were therefore based on limbs with available outcome data at each follow-up time point. Continuous variables were reported as mean ± standard deviation; categorical variables as counts and percentages. Exact 95% confidence intervals (CIs) for individual proportions were calculated using the Clopper–Pearson exact binomial method, whereas 95% CIs for risk differences, expressed as percentage points (pp) were calculated using the Newcombe method. Student’s *t*-test was used to assess group differences for continuous variables, and Fisher’s exact test was used for categorical variables. The sample size calculation was based on recurrence rates reported in the randomized study by de Roos et al., in which an absolute difference of approximately 35–40% was observed between surgical treatment and sclerotherapy at 2 years [15]. Assuming a 40% absolute difference in duplex recurrence rates between surgery and sclerotherapy, the required sample size to achieve 80% power at α = 0.05 was 20 limbs per group. A *p*-value < 0.05 was considered statistically significant. Statistical analyses were performed using RStudio version 2025.05.1+513 [20], R version 4.5.1 [21].

## 5. Results

### 5.1. Demographic Data

**Figure 1:**
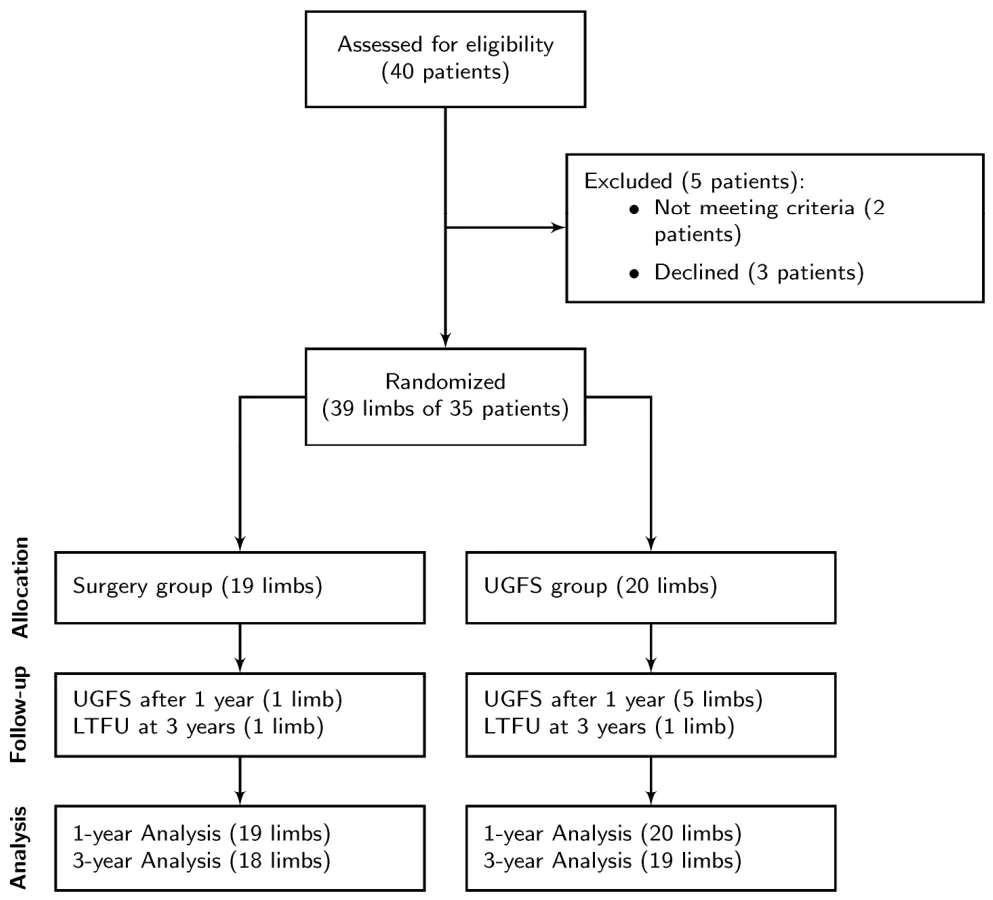
**Consolidated Standards of Reporting Trials** (CONSORT)-style flow diagram of patient and limb allocation, follow-up and analysis. LTFU = Lost to follow-up. UGFS = ultrasound-guided foam sclerotherapy. “UGFS after 1 year” denotes additional UGFS performed after completion of the 1-year follow-up assessment.

A total of 40 patients were eligible, of whom 5 were excluded (2 because of pregnancy and 3 because of patient preference). Between October 2006 and December 2008, 39 limbs of 35 patients were randomized and included in the analysis. The last documented 1-year and 3-year follow-up assessments were performed in January 2010 and May 2012, respectively. Two patients, one in each treatment group, withdrew consent and therefore did not attend the 3-year follow-up assessment (Figure 1). Four patients contributed two treated limbs each; in all four patients, one limb was allocated to surgery and the contralateral limb to UGFS.

Baseline characteristics were comparable between treatment groups, including sex, age, body mass index, ASV diameter, treatment side and highest clinical CEAP class. Most limbs were classified as CEAP C2 in both groups. Pain was the most frequently documented subjective symptom, followed by heaviness and cramps (Table 1).

Medication history was available for 30 patients; 13 (43%) were not taking any regular medication. The most common medication categories were venoactive drugs (7/30), antihypertensive or cardiovascular medication (7/30), and endocrine, bone-metabolism, or hematologic medication (5/30). No patient was receiving corticosteroids, antiplatelet therapy, heparin therapy or direct oral anticoagulants.

### 5.2. Ultrasound-Guided Foam Sclerotherapy Treatment Characteristics

For the initial UGFS treatment after study inclusion, ASV closure was achieved after one session in 10 limbs (50%), after two sessions in 6 limbs (30%), and after three sessions in 4 limbs (20%). The mean interval between subsequent injection sessions was 13 days (range, 5–46 days). The mean dose of polidocanol administered per session was 20.3 mg (range, 4–60 mg). The maximum polidocanol concentrations used per treatment session were 1%, 1.5%, 2%, and 3% in 17.6%, 11.8%, 58.8%, and 11.8% of sessions, respectively.

### 5.3. Follow-Up and Outcomes

At 3 years, the primary endpoint of duplex-detected recurrence in the ASV territory occurred in 6 of 18 limbs (33%; 95% CI, 13–59%) in the surgery group and 12 of 19 limbs (63%; 95% CI, 38–84%) in the UGFS group, corresponding to an absolute risk difference of 30 pp (95% CI −2–54 pp; *p* = 0.103). At 1 year, recurrence occurred in 2 of 19 limbs (11%; 95% CI, 1–33%) and 13 of 20 limbs (65%; 95% CI, 41–85%), respectively, corresponding to an absolute risk difference of 55 pp (95% CI 24–73 pp; *p* < 0.001; Table 2).

One limb in the surgery group underwent successful UGFS for NATV after completion of the 1-year follow-up. In the UGFS group, 5 of 13 limbs with duplex-detected recurrence at 1 year underwent additional sclerotherapy after the 1-year assessment: At 3 years, 2 showed complete occlusion, while 3 had a persistently incompetent ASV (Figure S1). All retreated limbs remained analyzed according to their original randomized treatment group.

In a sensitivity analysis addressing UGFS retreatment, a composite endpoint of duplex-detected ASV-territory recurrence or UGFS treatment after the 1-year assessment was used, with any retreatment classified as treatment failure. The composite endpoint occurred in 7 of 18 limbs in the surgery group and 14 of 19 limbs in the UGFS group (39% vs. 74%; absolute risk difference, 35 pp; 95% CI, 3 to 58 pp; *p* = 0.049).

A patient-level sensitivity analysis was performed by excluding 4 patients who contributed two treated limbs. After their exclusion, 31 patients with one treated limb each remained for analysis. At 1 year, ASV-territory recurrence occurred in 2 of 15 patients in the surgery group (13%) and 11 of 16 patients in the UGFS group (69%; *p* = 0.003). At 3 years, recurrence occurred in 6 of 14 patients after surgery (43%) and 11 of 15 patients after UGFS (73%; *p* = 0.139). Thus, the patient-level sensitivity analysis was consistent with the direction of the main limb-level analysis.

After 1 year, clinically visible varicose veins were present in 21% of limbs in the surgery group and 35% of limbs in the UGFS group (*p* = 0.481), and after 3 years, the proportion remained similar between groups, with 39% in the surgery group and 37% in the UGFS group (*p* > 0.9; Table 2).

At the 1-year follow-up, neovascularization was observed exclusively in the surgery group (26%), comprising 11% grade 1 and 16% grade 2 neovascularization. This difference between treatment groups was statistically significant (*p* = 0.020; Table 2). At 3 years, neovascularization remained more frequent after surgery than after UGFS (44% vs. 5%). In the surgery group, 6% of limbs demonstrated grade 1 and 39% grade 2 neovascularization, whereas in the UGFS group, 5% of limbs showed grade 2 and none showed grade 1. The difference between groups remained statistically significant (*p* = 0.009; Table 2).

No statistically significant difference in the incidence of new-onset incompetence of the GSV was observed between treatment groups during follow-up (Table 2). Specifically, two limbs, one in each group, demonstrated GSV incompetence at both the 1-year and 3-year follow-up. Three additional limbs developed new GSV incompetence at 3 years that had not been present at 1 year (1 in the UGFS group and 2 in the surgery group). One limb in the UGFS group showed new-onset GSV incompetence in addition to ASV-territory recurrence at 1 year, which was no longer detectable at 3 years after successful UGFS retreatment at 1 year.

## 6. Discussion

High-quality, head-to-head comparisons of therapeutic strategies for ASV incompetence are sparse. In the late 1990s, de Roos et al. conducted a randomized controlled trial comparing ambulatory phlebectomy with compression sclerotherapy for ASV incompetence with a follow-up of 2 years. Recurrence was more frequent in the compression sclerotherapy group than in the phlebectomy group at both 1 year and 2 years (12/48 vs. 1/48 and 18/48 vs. 1/48, respectively) [15]. In a cohort study from the United Kingdom, Bradbury et al. evaluated UGFS across different superficial veins, including ASV incompetence. Among patients treated for primary ASV incompetence (*n* = 93), recurrent reflux was detected in 3.6% of limbs during follow-up ranging from 8 to 64 months [11]. However, ASV-specific data in that study were derived from a broader cohort rather than from a randomized comparison.

The higher recurrence rate in the present trial compared with previous reports likely reflects differences in recurrence definition, follow-up structure and baseline vein anatomy. In Bradbury et al., ASV recanalization was reported when recurrent reflux led to repeat UGFS during follow-up, whereas the present trial recorded protocol-defined duplex-detected recurrence at fixed follow-up endpoints, irrespective of symptoms or retreatment [11]. Direct comparison with other studies is limited because most studies reported outcomes from mixed-vein cohorts without separate ASV-specific analysis [22,23,24,25,26,27,28,29]. A recent meta-analysis reported a pooled occlusion rate of 79.8% for liquid and foam sclerotherapy, based on two studies, whereas the above-mentioned phlebectomy study by de Roos, without high ligation, had a non-pooled occlusion of 97.9% at up to 24 months [14,15,28]. The quality of these studies was described as poor-to-moderate, follow-up as short and heterogeneity as substantial, thereby limiting firm comparative conclusions [14].

Hence, this randomized single-center trial adds evidence in an area where data are limited and provides a relatively long follow-up period of 3 years. Two distinct therapeutic approaches were compared. Surgery is a relatively resource-intensive intervention requiring operating room or treatment room availability and trained personnel, whereas UGFS is a minimally invasive, low-cost outpatient procedure that can be performed with readily available resources.

At the 1-year time point, surgery resulted in superior ASV-territory treatment success compared with UGFS. By 3 years, however, this difference had narrowed and was no longer statistically significant, which is in line with a previous report [6]. Although this difference did not reach statistical significance, the numerical difference may be clinically relevant. Two sensitivity analyses were performed. First, excluding patients with bilateral treatment did not substantially alter the findings, suggesting that bilateral cases had little influence on the overall results. However, the exploratory sensitivity analysis classifying additional UGFS as treatment failure revealed a borderline statistically significant difference favoring surgery, which suggests a possible advantage of surgery in terms of treatment durability, when retreatment is counted as treatment failure. Clinical recurrence rates did not differ significantly between groups at either follow-up time point. This divergence between duplex-defined outcomes and clinical recurrence has similarly been reported in other studies of GSV treatment, where EVTA or surgery typically achieved more durable occlusion than UGFS, whereas patient-reported and clinical outcomes often converged over time [30,31].

The type of recurrence differed by treatment modality in our study. Consistent with previous studies, neovascularization in the groin was observed after surgery but rarely after UGFS [32]. Notably, early duplex recurrence after surgery does not necessarily translate into clinical deterioration [33].

New-onset GSV incompetence was assessed during follow-up as an exploratory endpoint. Because concomitant GSV incompetence was an exclusion criterion at baseline, development of GSV incompetence during follow-up was considered a marker of possible progression of chronic superficial venous disease, altered superficial venous hemodynamics, or unmasking of previously borderline reflux. New-onset GSV incompetence was uncommon and did not differ significantly between treatment groups. We observed one patient in whom GSV competence was restored after successful UGFS treatment of the incompetent ASV. A plausible explanation for this observation is that, consistent with previous reports, elimination of tributary incompetence in selected cases can allow the GSV to regain competence [34,35].

Baseline vein anatomy may have negatively influenced outcomes in the UGFS group. The mean ASV diameter in this cohort was relatively large (~9 mm), whereas current varicose vein treatment guidelines recommend UGFS for saphenous trunks below a diameter of 6 mm [13,23]. Consequently, a proportion of UGFS-treated limbs in this study may have exceeded the diameter range in which sclerosing foam is most effective.

For patients with symptomatic varicose veins and axial reflux in the ASV, current varicose vein treatment guidelines gives a weak recommendation for EVTA or high ligation and stripping over physician-compounded UGFS when durable occlusion is the aim [12,13]. According to the German S2k guideline on the diagnosis and treatment of varicose veins, surgical high ligation and stripping is among the contemporary phlebologic treatment modalities [36]. Internationally, treatment methods used for truncal vein incompetence in clinical practice remain heterogeneous [37,38]. Although EVTA has been widely adopted in many regions, open vein surgery continues to be frequently performed and remains a standard treatment option in several health-care systems [39,40]. Consistent with this, recent survey data from certified German venous competence centers indicate that this procedure continues to be frequently performed, with 87.5% of centers reporting its use. In addition, reimbursement within the German statutory health insurance system remains largely centered on conventional surgery [41]. These factors highlight the continued clinical and health-care system relevance of the present study.

**Figure 2:**
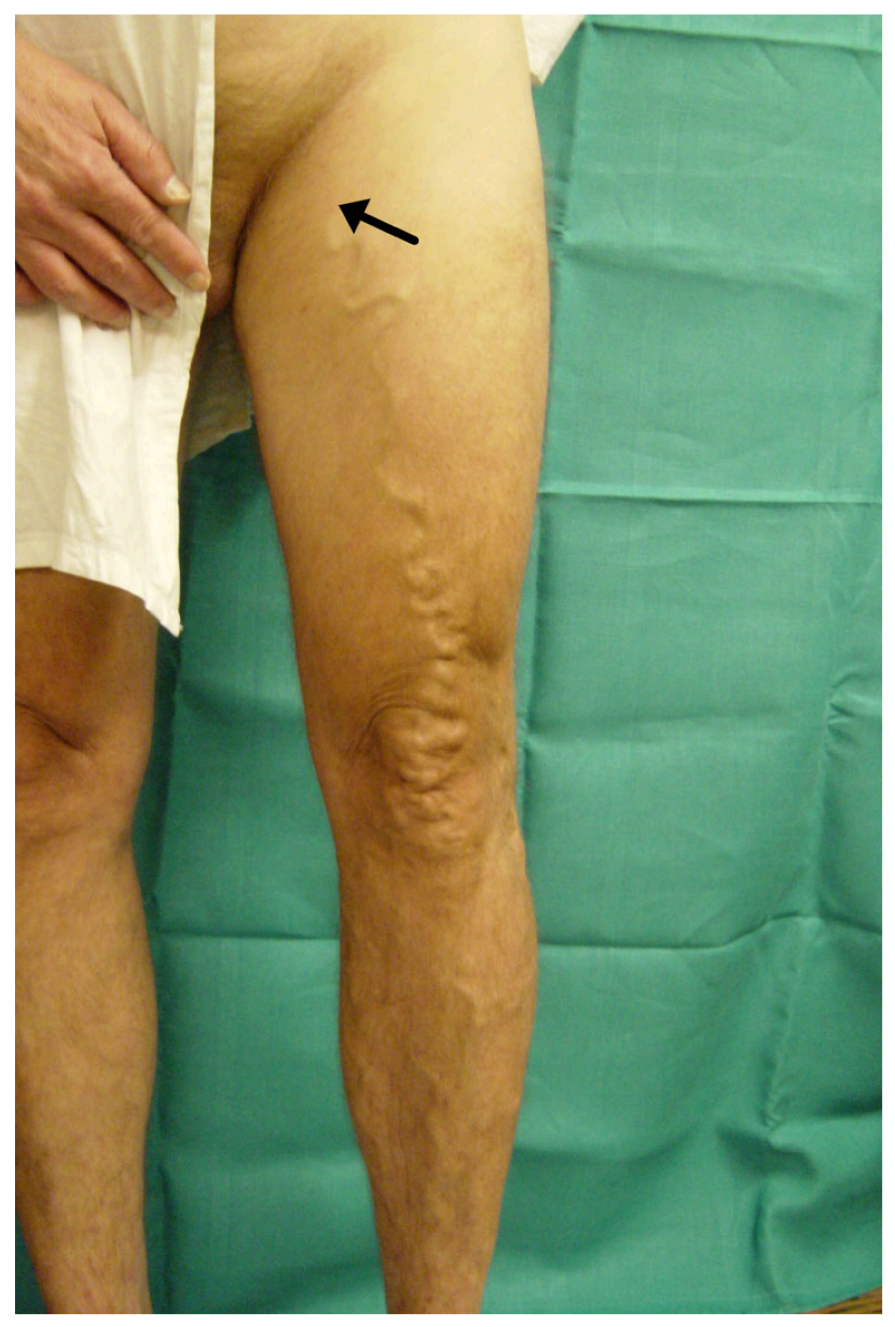
Clinical photograph illustrating pronounced tortuous varicosities in the anterior saphenous vein (ASV) territory, extending from the proximal thigh toward the knee. The arrow indicates the approximate location where the ASV exits its fascial compartment.

Furthermore, the relatively short length of the ASV can make cannulation for endothermal treatment technically challenging, leaving UGFS or surgery as the most viable alternatives in these patients (Figure 2). Therefore, evaluation of UGFS as a less invasive alternative to surgery was clinically reasonable, particularly because it is widely used for superficial venous incompetence and can be applied without preselection for suitability for EVTA. Alternatively, ambulatory selective varices ablation under local anesthesia (ASVAL) or “Conservatrice Hémodynamique de l’Insuffisance Veineuse en Ambulatoire” (CHIVA) may represent alternative treatment strategies in selected patients with ASV incompetence [42].

The strengths of this study include its randomized design, standardized duplex endpoints, and 3-year follow-up in a specific subgroup of limbs with isolated ASV incompetence. The small number of included limbs limits statistical power and the precision of effect estimates. Therefore, statistically non-significant differences should not be interpreted as evidence of equivalence. Given the wide CIs, clinically relevant differences cannot be excluded. Further limitations include the single-center setting, absence of blinded outcome assessment, and lack of prospectively collected quality-of-life or venous clinical severity scores. Another limitation is the treatment-specific definition of ASV-territory recurrence. Although these definitions were anatomically appropriate for each intervention, because recurrent disease may manifest differently depending on treatment modality, they may not capture fully comparable anatomical events. This should be considered when interpreting between-group differences. In addition, the statistically significant between-group difference observed at 1 year was a secondary finding and should be viewed accordingly. Although the study was approved by the institutional ethics committee and conducted according to the study protocol, prospective registration was not implemented as part of the original local study procedures. The trial was subsequently registered at ClinicalTrials.gov when the study was prepared for international publication. The long interval between study completion and manuscript submission was related to institutional changes affecting the senior author, which delayed manuscript preparation. The retrospective registration and absence of a separate prospective statistical analysis plan should be considered when interpreting the findings. CEAP clinical class was based on clinical examination [43]. Therefore, CEAP C0–C1 did not exclude ultrasound-detectable ASV-territory tributary disease. Adverse-event reporting is limited by the retrospective review of available study records. No procedure-related complications requiring specific treatment were documented. However, minor unrecorded adverse events cannot be excluded. The absence of an EVTA arm is an important limitation. Reported outcomes after EVTA for ASV incompetence are generally favorable, with high rates of anatomical closure [14]. EVTA was not included because a three-arm study would have required preselection of patients suitable for catheter-based treatment. Such patients may represent a selected subgroup and may not reflect the broad spectrum of ASV incompetence. ASV anatomy is highly variable, including differences in vein diameter, venous ectasia in proximity to the junction, tortuosity, length of the intrafascial segment, proximity to the superficial femoral artery, and association with refluxing inguinal lymph-node veins. These factors may determine whether EVTA is technically feasible [4]. In contrast, surgery and UGFS can be applied without such preselection. Including an EVTA arm would therefore have limited the representativeness of the study population and could have distorted the comparison between treatment strategies.

In this study, we used ligation of the GSV as the surgical method, reflecting common practice at the time of study initiation. Although current guidelines favor selective ASV treatment with preservation of the competent GSV [12,13], evidence regarding the optimal surgical approach remains limited. Accordingly, our findings may not be directly generalizable to GSV-preserving procedures, especially regarding long-term outcomes such as neovascularization and new-onset GSV incompetence.

In summary, surgery resulted in lower duplex-detected ASV-territory recurrence at 1 year. At 3 years, recurrence rates did not differ significantly between groups, although rates remained numerically higher in the UGFS group. Clinical recurrence rates were similar between groups, whereas groin neovascularization occurred more frequently after surgery. To optimize treatment results, the strategy for ASV treatment should therefore be based on shared decision-making, taking into account patient preferences regarding surgery, EVTA, expected recovery time and the possibility of multiple treatment sessions.

## Figures and Tables

**Table 1:** Baseline demographic and clinical characteristics for included limbs according to treatment group (surgery vs. UGFS). Continuous variables are presented as mean ± SD; categorical variables as counts (percentages). Differences between groups were assessed by Student’s *t*-test for continuous variables and Fisher’s exact test for categorical variables. Thirty-five patients contributed 39 treated limbs. Four patients contributed two treated limbs each; in all four patients, one limb was allocated to surgery and the contralateral limb to UGFS. Symptom data were available for 25 limbs: 11 in the surgery group and 14 in the UGFS group. Medication history was available for 30 patients and was missing for 5 patients each contributing 1 limb: 4 in the surgery group and 1 in the UGFS group. Abbreviations: UGFS, ultrasound-guided foam sclerotherapy; SD, standard deviation; BMI, body mass index; ASV, anterior saphenous vein; CEAP, Clinical–Etiological–Anatomical–Pathophysiological classification.

Characteristic	Surgery *N* = 19 ^1^	UGFS *N* = 20 ^1^	*p*-Value ^2^
Sex			0.6
female	17 (89%)	19 (95%)	
male	2 (11%)	1 (5.0%)	
Age at treatment, years	52 ± 16	48 ± 14	0.5
BMI, kg/m^2^	26.4 ± 4.3	27.1 ± 5.0	0.7
ASV diameter, mm	8.97 ± 3.62	8.90 ± 2.34	>0.9
Side			0.5
right	6 (32%)	9 (45%)	
left	13 (68%)	11 (55%)	
Highest CEAP C			0.7
C0	1 (5.3%)	3 (15%)	
C1	2 (11%)	1 (5.0%)	
C2	12 (63%)	11 (55%)	
C3	2 (11%)	4 (20%)	
C4	2 (11%)	1 (5.0%)	
Pain	5 (45%)	11 (79%)	0.1
Heaviness	3 (27%)	4 (29%)	>0.9
Cramps	1 (9.1%)	1 (7.1%)	>0.9

^1^ *n* (%); mean ± SD. ^2^ Fisher’s exact test; two-sample *t*-test. Symptom data were available for 25 limbs: 11 in the surgery group and 14 in the UGFS group.

**Table 2:** Clinical outcomes at 1- and 3-year follow-up by treatment group. Values are *n* (%). *p*-values from Fisher’s exact test. Abbreviations: UGFS, ultrasound-guided foam sclerotherapy; ASV, anterior saphenous vein; GSV, great saphenous vein.

	1 Year			3 Years		
Outcome	Surgery *N* = 19 ^1^	UGFS *N* = 20 ^1^	*p*-Value ^2^	Surgery *N* = 18 ^1^	UGFS *N* = 19 ^1^	*p*-Value ^2^
ASV-territory recurrence			**<0.001**			0.103
yes	2 (11%)	13 (65%)		6 (33%)	12 (63%)	
no	17 (89%)	7 (35%)		12 (67%)	7 (37%)	
Clinical recurrence			0.481			>0.9
yes	4 (21%)	7 (35%)		7 (39%)	7 (37%)	
no	15 (79%)	13 (65%)		11 (61%)	12 (63%)	
Neovascularization			**0.020**			**0.009**
Grade 0	14 (74%)	20 (100%)		10 (56%)	18 (95%)	
Grade 1	2 (11%)	0 (0%)		1 (5.6%)	0 (0%)	
Grade 2	3 (16%)	0 (0%)		7 (39%)	1 (5.3%)	
GSV incompetence			>0.9			0.660
yes	1 (5.3%)	2 (10%)		3 (17%)	2 (11%)	
no	18 (95%)	18 (90%)		15 (83%)	17 (89%)	

^1^ *n* (%). ^2^ Fisher’s exact test. Statistically significant *p*-values (*p* < 0.05) are highlighted in bold.

## Data Availability

Data: supporting the findings of this study are provided in the Supplementary Materials. Additional information is available from the corresponding author upon reasonable request.

## Supplementary Materials

The following supporting information can be downloaded at: https://www.mdpi.com/article/10.3390/jcm15218343/s1, Figure S1: Changes in duplex-detected ASV-territory recurrence status between the 1-year and 3-year follow-up assessments, stratified by treatment group. Red flows indicate no additional treatment, whereas blue flows indicate additional UGFS after the 1-year assessment. LTFU = Lost to follow-up.

## Abbreviations

ASV	Anterior saphenous vein.
ASVAL	Ambulatory selective varices ablation under local anesthesia.
CEAP	Clinical-Etiological-Anatomical-Pathophysiological classification.
CHIVA	Cure Conservatrice et Hémodynamique de l’Insuffisance Veineuse en Ambulatoire.
CI	Confidence interval.
CONSORT	Consolidated Standards of Reporting Trials
EVTA	Endovenous thermal ablation.
GSV	Great saphenous vein.
LTFU	Lost to follow-up
NATV	new anterior thigh varicosities connected to the previous saphenofemoral junction.
pp	percentage points.
SD	Standard deviation
UGFS	Ultrasound-guided foam sclerotherapy.
UIP	International Union of Phlebology
