# Supplementary material for: Surgery Versus Ultrasound-Guided Foam Sclerotherapy for the Treatment of Isolated Anterior Saphenous Vein Incompetence: A Single-Center Randomized Trial
Source: J Clin Med. 2026 Oct 26;15(21):8343. doi: 10.3390/jcm15218343 (PMC13764045; doi:10.3390/jcm15218343)
Supplement: Supplementary file 1 [file jcm-15-08343-s001.zip › jcm-4550967-supplementary.pdf]

Figure S1

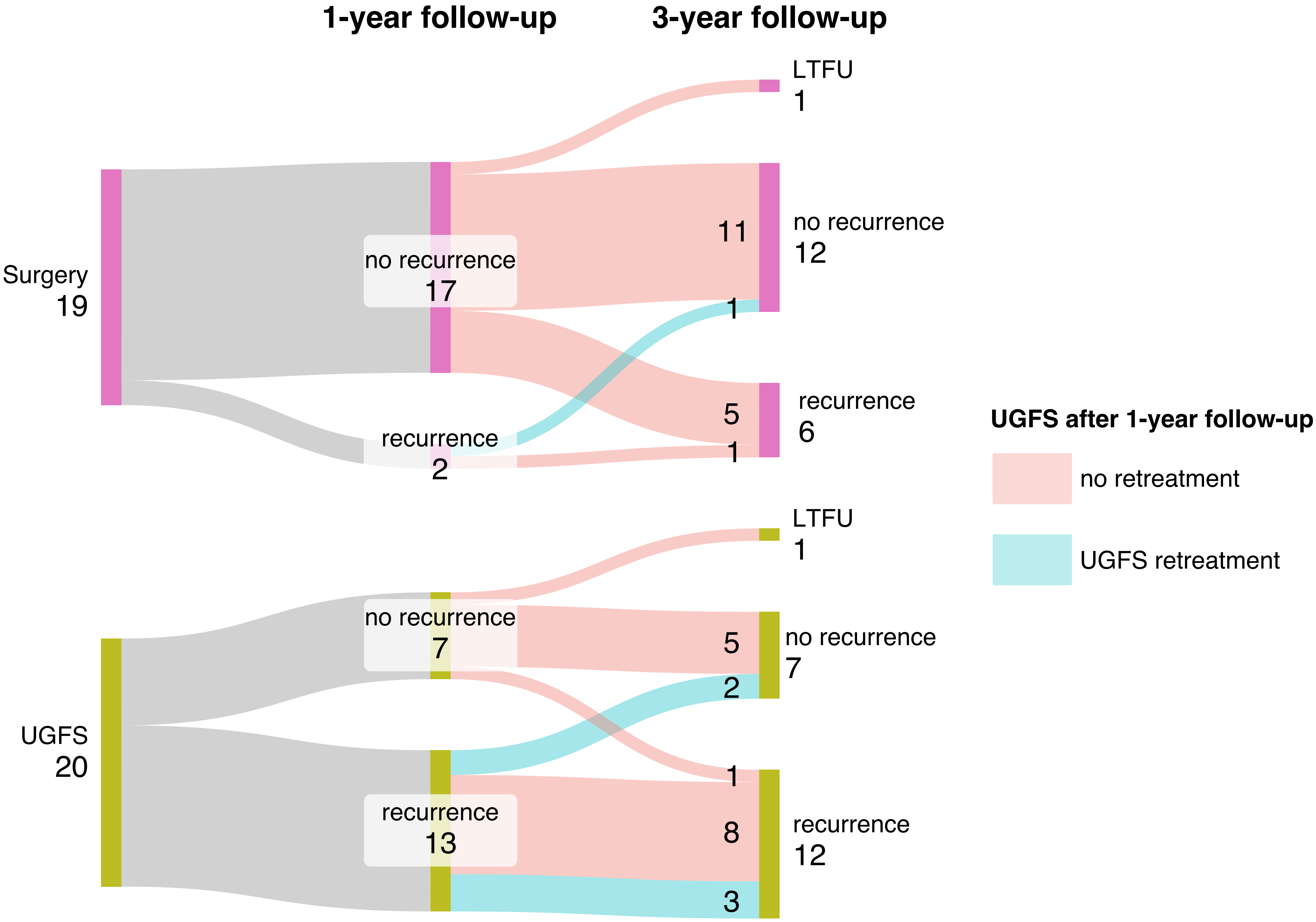

1-year follow-up
3-year follow-up
Surgery
19
no recurrence
17
recurrence
2
LTFU
1
11
no recurrence
12
1
5
recurrence
6
1
UGFS
20
no recurrence
7
recurrence
13
LTFU
1
5
no recurrence
7
2
1
8
recurrence
12
3
UGFS after 1-year follow-up
no retreatment
UGFS retreatment
